# Periprosthetic fractures around chronically infected total hip arthroplasties: a case series and pragmatic two-stage treatment framework

**DOI:** 10.5194/jbji-11-547-2026

**Published:** 2026-08-25

**Authors:** Georges F. Vles, Willem-Jan Metsemakers, Laurens Aerden, Jan Geurts, Oliver Borens, Stijn Ghijselings

**Affiliations:** 1 Department of Orthopaedic Surgery, University Hospitals Leuven, Leuven, 3000, Belgium; 2 Institute for Orthopaedic Research and Training (IORT), KU Leuven, Leuven, 3000, Belgium; 3 Department of Development and Regeneration, KU Leuven, Leuven, 3000, Belgium; 4 Department of Trauma Surgery, University Hospitals Leuven, Leuven, 3000, Belgium; 5 Department of Orthopaedic Surgery, Research School CAPHRI, Maastricht University Medical Centre, Maastricht, 6229 HX, the Netherlands; 6 Department of Orthopaedic Surgery, Clinique Bois Cerf, Hirslanden Group, Lausanne, 1006, Switzerland

## Abstract

Three periprosthetic femoral fractures around chronically infected total hip arthroplasties (THAs) were treated according to a pragmatic two-stage treatment framework. Infection control and fracture healing were achieved. This may serve as a reference for surgeons confronted with these complex and technically demanding situations.

## 1 Introduction

The annual number of total hip arthroplasties (THAs) is projected to increase substantially over the coming decades, driven by population growth and rising utilization rates (Pabinger et al., 2018; Shichman et al., 2023). Although revision rates per implant have gradually declined – largely due to improvements in bearing surfaces and implant fixation – the absolute number of revision procedures continues to rise in parallel with the increasing volume of primary THAs (Achakri et al., 2023; Smith et al., 2023).

Periprosthetic joint infection (PJI) and postoperative periprosthetic fracture (PPFx) remain two of the most devastating complications following THA. However, the true incidence of both complications is likely underestimated in registry-based studies. For PJI, underestimation results from incomplete reporting and the failure to update the preoperative diagnosis based on intraoperative findings, including microbiological cultures obtained during revision surgery (Gundtoft et al., 2015; Lutro et al., 2024). For PPFx, the underestimation is due to joint registries capturing only cases requiring stem revision. As more than half of PPFx cases are treated without stem revision, a substantial proportion remains unrecorded, yielding an estimated true incidence of 0.92 per 1000 prosthesis years (Lamb et al., 2024). The absolute number of patients developing PJI or PPFx is expected to increase substantially in the coming decades, driven by the rising volume of primary and revision arthroplasty procedures, an ageing population with increasing medical comorbidity, and the growing number of individuals living with prosthetic joints (Pivec et al., 2015; Kurtz et al., 2018; Smith et al., 2023).

Although both PJI and PPFx are well-described complications in isolation, their coexistence represents a rare and particularly challenging clinical scenario (van den Kieboom et al., 2021; Shah et al., 2016; Chevillotte et al., 2009; Lazic et al., 2022). Importantly, a distinction must be made between PPFx occurring around a chronically infected THA and acute PJI (or fracture-related infection, FRI) developing as a complication of revision surgery for PPFx. These entities differ fundamentally in terms of pathophysiology, timing, and treatment strategy. The presence of a mature biofilm in chronic infection is a key factor influencing treatment decisions and distinguishes these cases from acute postoperative infections. Fractures around chronically infected implants typically occur in the setting of longstanding infection and septic loosening, whereas infections following fracture surgery are generally acute postoperative events. Given that implant loosening is a well-established risk factor for PPFx, it is plausible that septic loosening further increases fracture susceptibility, although robust data on this relationship remain limited (Shah et al., 2016).

Evidence-based guidelines exist for the management of PPFx (Patsiogiannis et al., 2021), PJI (Izakovicova et al., 2019) and FRI (Metsemakers et al., 2020). However, integrating these principles of fracture stabilization, infection eradication, and definitive reconstruction in this often frail patient population is challenging, and expected outcomes remain poorly defined. To date, no standardized treatment algorithm has been established for this complex overlap scenario.

The purpose of this case series is to describe the outcomes of three clinical cases with PPFx around chronically infected THAs treated using a consistent, staged revision approach. By reporting clinical, radiographic, and infection-related outcomes, we aim to contribute to the limited body of evidence in this field and to propose a pragmatic treatment framework for managing these challenging cases.

## 2 Cases

### 2.1 Case 1

#### 2.1.1 Background

A 74-year-old woman (BMI 28.3, ASA III, Charlson Comorbidity Index 7) sustained a right femoral neck fracture in 2019, for which she underwent THA at her local hospital. The postoperative course was complicated by an *Escherichia coli* (ESBL+) PJI, treated sequentially with open wash-out without exchange of mobile parts; then debridement, antibiotics, and implant retention (DAIR); and ultimately a three-stage exchange.

**Figure 1:**
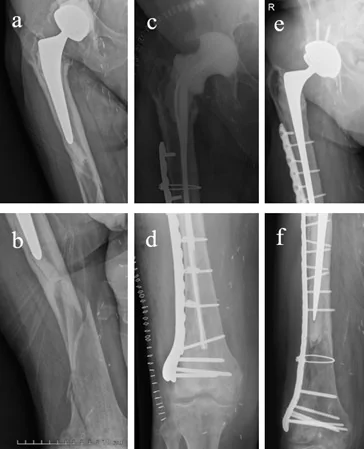
**(a–b)** Vancouver C PPFx, **(c–d)** situation after first stage, **(e–f)** situation after second stage.

She was lost to follow-up until 2022, when she presented after a low-energy fall resulting in a PPFx. The patient reported persistent thigh pain since her reimplantation, particularly during weight bearing. Conventional radiographs demonstrated a Vancouver type C PPFx with evidence of pre-existent loosening of the cemented stem (Fig. 1a–b).

Relevant comorbidities included a previous (redo) gastric bypass, coronary artery bypass grafting (CABG), and peripheral arterial disease (PAD) treated with bypass grafts and stents.

Fluoroscopy-guided hip aspiration under sedation revealed a synovial white blood cell count of 15 880 × 10^6^ L^−1^ with 97 % polymorphonuclear neutrophils and a positive alpha-defensin point-of-care test, fulfilling the EBJIS criteria for a PJI. PCR was positive for *Staphylococcus* spp., and culture subsequently confirmed methicillin-resistant *S. epidermidis* (MRSE).

After multidisciplinary team (MDT) discussion and shared decision-making with the patient, a two-stage revision strategy was selected.

#### 2.1.2 First stage

Prior to surgery, a custom-made spacer was prepared by lengthening a prefabricated Tecres spacer (vancomycin + gentamicin; Tecres S.p.A., Verona, Italy) in order to bypass the fracture and also to incorporate meropenem, given the patient's history of ESBL-producing *E. coli* PJI.

An extensile posterolateral and subvastus approach was performed to explant the THA, followed by extensive debridement and lavage.

During the subsequent “clean” phase (Vles et al., 2022), the PPFx was stabilized using cerclage wiring and a plate–screw construct (VA LCP distal femur plate; DePuy Synthes, Raynham, MA, USA). The custom-made spacer was then implanted (Fig. 1c–d).

Two out of 10 intraoperative tissue cultures were positive for MRSE, as was the sonication sample. Empirical broad-spectrum antibiotics (vancomycin and piperacillin–tazobactam) were initiated perioperatively. Piperacollin–tazobactam was stopped on postoperative day 3, and vancomycin was de-escalated to oral minocycline (100 mg twice daily) on postoperative day 14. Wound healing was uneventful, and C-reactive protein (CRP) normalized to 1.1 mg L^−1^.

#### 2.1.3 Second stage

Ten weeks postoperatively, the second-stage revision was performed.

In the initial “dirty” phase, the spacer and fixation hardware were removed, followed by repeat debridement and thorough lavage. The surrounding tissues appeared healthy, and the fracture demonstrated radiological and intraoperative signs of healing.

During the subsequent “clean” phase, new plate–screw fixation (Zimmer Biomet NCB plate; Zimmer Biomet, Warsaw, IN, USA) was applied to reinforce the femur and to reduce the risk of recurrent PPFx. Definitive reconstruction consisted of a trabecular metal shell with a cemented dual mobility cup (Avantage; Zimmer Biomet, Warsaw, IN, USA) and a long cemented femoral stem (CPT; Zimmer Biomet, Warsaw, IN, USA) (Fig. 1e–f).

Perioperative antibiotics were escalated to vancomycin and subsequently transitioned to oral minocycline (100 mg twice daily) and rifampin (450 mg twice daily) on postoperative day 7 for 6 weeks postoperatively. All intraoperative cultures obtained at the second stage remained negative. The postoperative course was uncomplicated.

#### 2.1.4 Outcome at final follow-up

At final follow-up, 3.5 years after the second-stage procedure, radiographs and CT imaging demonstrated complete fracture healing. The PJI appeared to have been eradicated, with normalization of CRP (1.6 mg L^−1^), complete wound healing, and no evidence of osteolysis. The patient even reported improved mobility compared with her status prior to sustaining the PPFx.

### 2.2 Case 2

#### 2.2.1 Background

A 56-year-old man (BMI 19, ASA III, Charlson Comorbidity Index 3) with a history of avascular necrosis of the right femoral head underwent hip resurfacing at his local hospital in 2007. This was complicated by early PJI, treated with open wash-out without exchange of mobile parts. In 2020, he underwent a two-stage revision for chronic PJI due to *S. aureus* and *Pseudomonas aeruginosa*.

**Figure 2:**
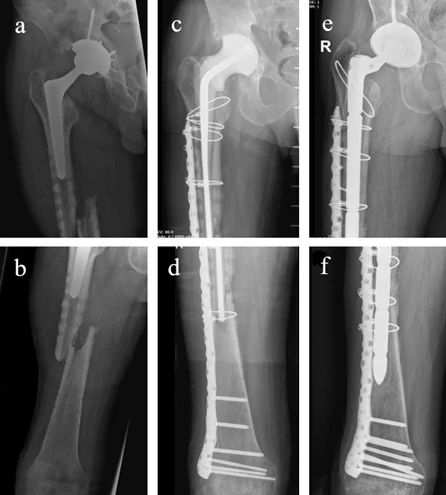
**(a–b)** Vancouver C PPFx, **(c–d)** situation after first stage, **(e–f)** situation after second stage.

In 2023, he sustained a PPFx, managed with plate–screw osteosynthesis, which failed due to non-union (Fig. 2a–b).

Cultures taken during plate removal 1 year after fracture fixation again grew *P. aeruginosa*, prompting referral to our unit.

Past medical history included CABG and chronic alcohol use.

Fluoroscopy-guided arthrocentesis under sedation revealed synovial WBC of 59 800 × 10^6^ L^−1^ with 99 % PMNs confirming PJI according to the EBJIS criteria. Cultures remained negative, possibly as a consequence of ongoing piperacillin–tazobactam therapy, which had been initiated at the referring hospital.

Following MDT discussion and shared decision-making, a two-stage revision strategy was chosen.

#### 2.2.2 First Stage

In the initial “dirty” phase, an extensile posterolateral approach was used and frank pus was encountered both along the plate as well as within the joint. The plate and cup were removed easily; however, stem extraction required an extended trochanteric osteotomy (ETO) as it was impossible to provide sufficient counterforce on the proximal femur to allow antegrade stem extraction.

In the “clean” phase, the fracture was stabilized with cerclage wires and a plate–screw construct (Zimmer Biomet NCB plate; Zimmer Biomet, Warsaw, IN, USA). A long antibiotic-loaded cement spacer (vancomycin + gentamicin; Tecres S.p.A., Verona, Italy) was implanted (Fig. 2c–d).

Three out of 15 intraoperative cultures – taken from both the plate and joint space, including sonication of the THA components – grew *P. aeruginosa*. Broad-spectrum antibiotics (piperacillin–tazobactam) were started and later de-escalated to oral levofloxacin (500 mg BID) on postoperative day 11, continued for 8 weeks. Wound healing was uncomplicated, although CRP remained mildly elevated ( ∼ 40 mg L^−1^).

#### 2.2.3 Second stage

At the second-stage “dirty” phase, the spacer and hardware were removed, and repeat debridement was performed. Tissue appeared healthy, but neither the fracture nor the ETO had healed.

In the “clean” phase, the femoral non-union was treated with a new plate–screw construct (Zimmer Biomet NCB plate; Zimmer Biomet, Warsaw, IN, USA), and reconstruction of the hip was completed with an uncemented dual mobility cup (G7; Zimmer Biomet, Warsaw, IN, USA) and a modular revision stem (ILS 250; Zimmer Biomet, Warsaw, IN, USA) (Fig. 2e–f).

Perioperative antibiotics were again escalated to vancomycin and piperacillin–tazobactam at induction. While intraoperative cultures were negative for *P. aeruginosa*, six samples returned positive for *Candida albicans*. Piperacillin–tazobactam was continued for an additional 6 weeks via outpatient parenteral antibiotic therapy (OPAT) to complete the course for *P. aeruginosa* (Thijs et al., 2022). Concurrently, antifungal therapy with fluconazole (400 mg once daily) was initiated for *C. albicans* and continued for 6 months. The postoperative course was uneventful.

#### 2.2.4 Final follow-up

At final follow-up, 2 years after the second-stage procedure, radiographs and CT imaging demonstrated complete fracture healing. The PJI appeared to have been eradicated, with normalization of CRP (1.8 mg L^−1^), complete wound healing, and no evidence of osteolysis. No subsidence of the ILS stem occurred. Nevertheless, PROMs remained poor: HOOS: pain 35, symptoms 30, activities 25, functioning 0, and QoL 25.

### 2.3 Case 3

#### 2.3.1 Setting

A 76-year-old woman (BMI 27, ASA II, Charlson Comorbidity Index 3) sustained a right femoral neck fracture 3 years prior, for which she underwent an uncemented THA via a direct anterior approach (DAA) at her local hospital. Her medical history was significant for a complicated postoperative course following hysterectomy, a left THA that had already been revised for a PPFx, atrial fibrillation, and a prior C1–C2 fusion for a dens fracture.

**Figure 3:**
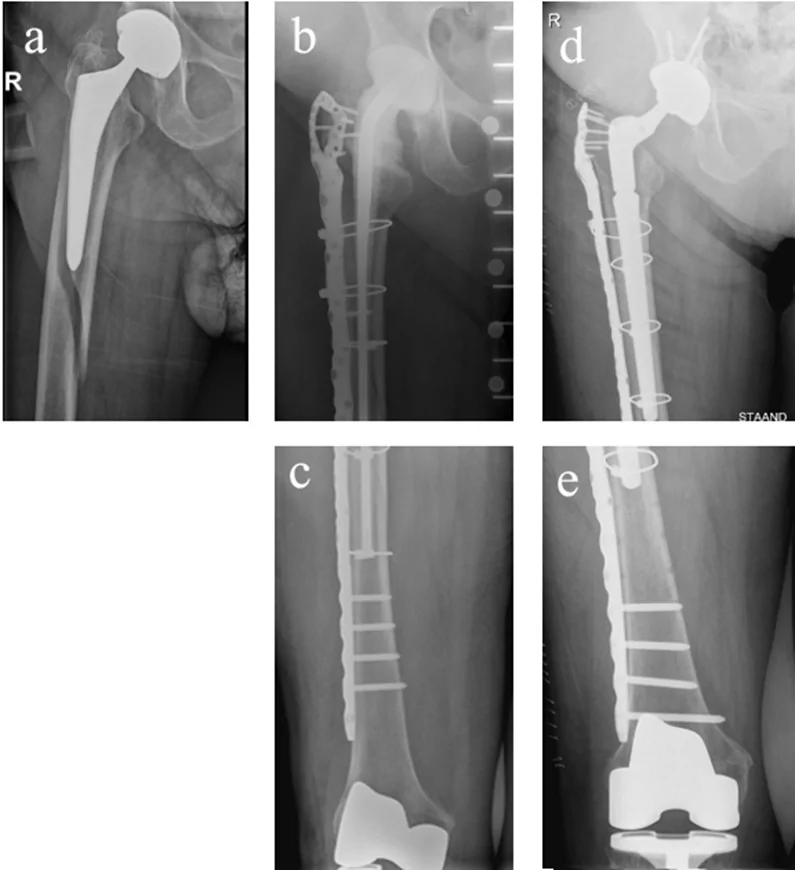
**(a)** Vancouver B2 PPFx, **(b–c)** situation after first stage, **(d–e)** situation after second stage.

Recently, she developed a cutaneous fistula at the site of her previous DAA incision. Shortly thereafter, she sustained a Vancouver B2 PPFx around the right THA following a fall and was referred to our orthopaedic department for further management (Fig. 3a).

Following MDT discussion and shared decision-making with the patient, a two-stage revision strategy was adopted.

#### 2.3.2 First stage

During the “dirty” phase, the original DAA incision was utilized to trace and excise the fistula down to the implant. An extensile posterolateral and subvastus approach was then used to explant the THA, and perform thorough debridement and lavage of the bone and soft tissues.

In the subsequent “clean” phase, the fracture was stabilized with cerclage wires and a plate–screw osteosynthesis construct with trochanteric extension (NCB plate; Zimmer Biomet, Warsaw, IN, USA). A long, antibiotic-loaded cement spacer (vancomycin + gentamicin; Tecres S.p.A., Verona, Italy) was implanted (Fig. 3b–c). Two out of 15 tissue samples returned positive for *Cutibacterium avidum*, as did the sonication sample. Empirical broad-spectrum antibiotics (vancomycin and piperacillin–tazobactam) were initiated at induction. Piperacillin–tazobactam was stopped on postoperative day 3, and vancomycin was de-escalated to oral amoxicillin (1 g four times daily) based on sensitivities on postoperative day 14. Wound healing was uncomplicated, and C-reactive protein (CRP) levels decreased to 9.6 mg L^−1^. 

#### 2.3.3 Second stage

Eight weeks postoperatively, the second-stage procedure was performed. In the “dirty” phase, the spacer and hardware were removed, and repeat debridement and lavage were conducted. The tissues appeared healthy, and the fracture demonstrated signs of healing. During the “clean” phase, new plate–screw fixation was applied to reinforce the femur and mitigate the risk of future PPFx. The definitive implant included an uncemented dual mobility acetabular component (G7; Zimmer Biomet, Warsaw, IN, USA) secured with screws; and a modular fluted, tapered, titanium revision femoral stem (ARCOS 150; Zimmer Biomet, Warsaw, IN, USA) (Fig. 3d–e).

Antibiotic prophylaxis with intravenous ceftriaxone was administered at induction and switched back to oral amoxicillin on postoperative day 4. All intraoperative cultures taken during the second stage remained negative. The postoperative course was largely uneventful except for surgical evacuation of a small haematoma. The patient completed a total of 12 weeks of antibiotic therapy, initiated at the time of the first-stage procedure.

#### 2.3.4 Outcome at final follow-up

At final follow-up in clinic, 7 months after the second-stage procedure, the patient was doing well and reported no problems with regard to her THA. She refused further laboratory and radiological investigations. Over-the-phone follow-up 1 year after reimplantation, she confirmed no problems had come up. PROMs were excellent: HOOS: pain 100, symptoms 90, activities 100, functioning 100, and QoL 100).

## 3 Discussion

**Figure 4:**
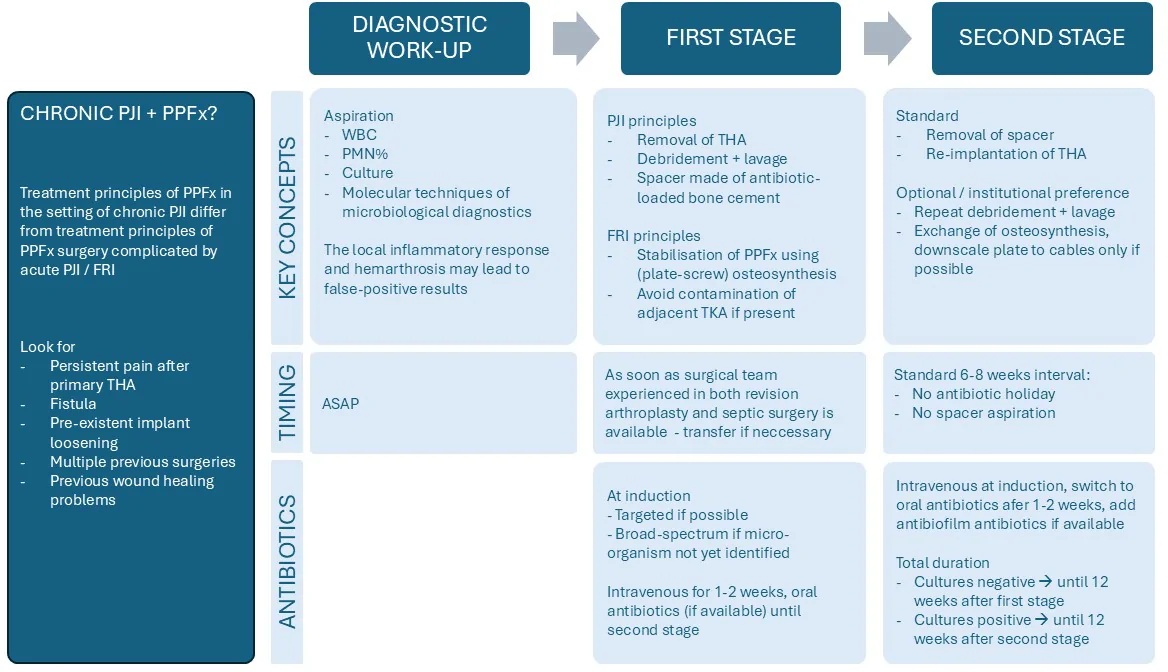
Treatment framework currently in use at our institution.

We report three complex cases of PPFx around chronically infected THAs, all managed using a consistent two-stage revision strategy (Fig. 4). Despite the inherent technical challenges, fracture healing and infection control according to the Delphi consensus was achieved (Diaz-Ledezma et al., 2013). Inherent to the pathology and patient characteristics, functional outcomes remained variable.

Diagnosis of PJI in the setting of a PPFx can be challenging. While the presence of a sinus tract provides a clear diagnostic indicator (case 3), clinical suspicion should also be raised in patients with pre-existing implant loosening (case 1) or a history of multiple prior surgeries (case 2), prompting joint aspiration. Interpretation of synovial fluid parameters, however, including leukocyte count and differential, requires caution. Apart from wear and underlying crystallopathy, hemarthrosis and the resulting local inflammatory response may lead to false-positive results (van den Kieboom et al., 2021; Bedair et al., 2011). This will particularly be true when the fracture extends into the joint space. Noting whether the aspirate is serous or sanguineous is important. At our institution, the initial 1 mL of synovial aspirate is routinely allocated for leukocyte count and differential before additional fluid is aspirated. This practice is intended to minimize blood contamination of the sample and to preserve the diagnostic accuracy of synovial fluid analysis. Molecular techniques of microbiological diagnostics may provide additional diagnostic value in this context.

The timing of intervention is a critical consideration. Delays caused by awaiting the growth of low-virulence microorganisms may potentially compromise outcomes. However, recent studies (van Laarhoven et al., 2021; Aerden et al., 2026) did not demonstrate a clear correlation between the time interval from trauma to definitive surgery and patient outcomes, including mortality. When necessary, postponing the procedure until it can be performed by a surgical team experienced in both revision arthroplasty and septic surgery appears to be a safe and prudent strategy.

The management of these cases requires adherence to the fundamental principles of both PJI and FRI treatment.

PJI principles include prompt initiation of (targeted) antibiotics (at induction), thorough surgical debridement and lavage, and complete removal of foreign material. Extraction of loose implants is typically straightforward; however, the removal of well-fixed stems can be technically demanding, particularly in the setting of a fractured or difficult-to-manipulate femur. In cases where adequate counterforce cannot be applied, an ETO may be necessary to extract stems that would otherwise be removable from the top (case 2).

FRI principles emphasize achieving stable fixation while preserving bone stock and biological potential for healing. Although intramedullary spacers may provide some stability, additional measures are often required to achieve sufficient stability to prevent ongoing soft tissue compromise, disrupted revascularization, and bone osteolysis, as described by Stephen Perren (Foster et al., 2021). During the first stage, it is advisable to preserve some screw holes in the plate construct or to take the shortest possible plate without compromising stability to allow optimal fixation options during the second-stage reconstruction. Consideration of adjacent prosthetic joints is also essential, in this case TKAs, as the risk of metachronous infection is reported to be as high as 20 % (Akkaya et al., 2023).

The interval between stages must be carefully individualized. Prolonged delays before reimplantation may increase the risk of septic failure, while premature reimplantation may compromise fracture healing. In our practice, a window of approximately 6 weeks without an antibiotic-free interval is generally applied; however, in the context of PPFx, we may extend this period to allow the fracture to reach more advanced stages of healing. Additional factors that should be considered include wound healing status, the trend of inflammatory markers such as CRP, the patient's ability to tolerate the spacer, and radiographic evidence of acetabular bone erosion or other spacer-related complications.

The fracture location, healing potential, isthmus integrity, bone quality, and patient age guide the selection of femoral stems. Three different revision stems were used in the cases described above. We typically revise plate–screw osteosyntheses during the second stage in the same manner that the spacer is exchanged for the definitive implant. Conceptually, we consider the second stage a “single-stage exchange of the spacer”, involving repeat debridement and lavage, the division of the procedure into dirty and clean phases, and the complete replacement of all components. This approach is currently based on expert opinion rather than robust scientific evidence and remains at the surgeon's discretion. Some surgeons advocate leaving the osteosynthesis from the first stage in situ as it was implanted under antibiotic coverage, and replacing it will increase operating time, blood loss, and soft tissue dissection (Diot et al., 2026). Our main concern is the potential introduction of microorganisms during the first-stage procedure, which is often a prolonged and technically demanding surgical intervention. If present, these organisms may not have been adequately covered by the targeted antimicrobial therapy directed against the presumed causative pathogen. A substantial proportion of patients has been reported to have one or more positive cultures for methicillin-resistant *S. epidermidis* (MRSE) at the time of second-stage reimplantation, although the clinical significance of these findings remains uncertain (Theil et al., 2020). During the second stage, the fracture has already been reduced (and ideally partially healed), and the majority of the surgical approach has already been performed, making plate exchange a relatively straightforward procedure compared with performing the osteosynthesis during the first stage.

Finally, the role of single-stage management in this setting remains uncertain. Although data are limited, it may be feasible in selected cases. Scenarios in which this approach might be considered include situations where infection is identified only after initial fracture fixation and the patient achieves satisfactory outcomes, or in frail hosts where fracture stabilization is the primary concern and chronic PJI (or contamination of the implant) can be managed with suppressive antibiotics or even tolerated without.

This case series has several limitations, many of which are inherent to the rarity and complexity of the condition under investigation. The sample size is small, and there is substantial heterogeneity with respect to the causative pathogens, fracture patterns, and implant characteristics. Furthermore, the absence of a control group precludes comparative analyses, and the proposed treatment considerations are based primarily on expert opinion rather than comparative evidence. These limitations underscore the need for large musculoskeletal infection centres to establish and share prospective databases, while international infection societies should collaborate to develop consensus-based treatment algorithms.

In conclusion, in this case series, an expert-opinion-based framework for PPFx associated with chronically infected THAs is illustrated. This may serve as a reference for surgeons confronted with these complex and technically demanding situations.

## Data Availability

No data sets were used in this article.
